# The Natural History and Diseases of the Human Teeth

**Published:** 1846-03

**Authors:** 


### Bibliographical Notices.

*The Natural History and Diseases of the Human Teeth.* By JOSEPH FOX, M. R. C. S. L., Member of the Society of Medicine, Paris; Lecturer on Structure and Diseases of the Teeth at Guy's Hospital, &c. First American from the third London Edition, Remodeled, with an Introduction and numerous additions, by CHAPIN A. HARRIS, M. D., D. D. S., Professor of Practical Dentistry, and Dental Pathology, in the Baltimore College of Dental Surgery; author of Principles and Practice of Dental Surgery, &c. &c. Illustrated with thirty plates. Philadelphia: EDWARD BARRINGTON and GEO. D. HASWELL, 1846, pp. 440.

We are indebted to the publishers for a copy of the above work. We greatly regret not receiving it in time, to give it any adequate notice in the present number of the American Journal of Dental Science. The original work of Mr. Fox, has been before the public for more than forty years, and no commendation from us, would add any thing to the high estimation in which it has ever been held. Very few, if any dental writers, have enjoyed greater advantages for obtaining correct information upon most of the topics connected with this subject, than has Mr. Fox, and few, certainly, have been more successful in imparting the result of his profound researches to his fellows. His work, notwithstanding it has been written nearly half a century, is still sought and admired, not only for the valuable information it contains, but, as being one of the best specimens of dental literature. But since Mr. Fox's day, many valuable acquisitions have been made, not only in the mechanical, but in the scientific department of this useful branch of the healing art. Since his time, new facts have been brought to light, new principles discovered, old theories demolished, and new ones, more compatible with facts and sound philosophy have been substituted.

In no department of the dental art, however, have so great changes been wrought as in the mechanical. The accomplished surgeon dentist, of 1803, so far as the mechanical department is concerned, would now, be but a backward student.

To make, therefore, such a work as that of Mr. Fox's well adapted to instruct the student in dentistry, at the present time, many corrections are necessary, and many additions are required. And we most heartily rejoice, that this task has been undertaken by one so well prepared to do the subject justice. Than Dr. Harris, no man is better prepared to correct all that may need correcting, and to add every modern improvement, which could in the least enhance its value to the student. And, so far as we have been enabled to examine this work, we are prepared to express our belief, that it is one which will add new laurels to the many which have hitherto been justly awarded to him as a dental writer.

Nor have the publishers done themselves less credit in executing it, its typographical neatness and accuracy, and the general good taste displayed throughout, do them great credit.

On the whole, considering the vast amount of practical information it contains, the judicious arrangement of its subjects, and last, though not least, the great number of valuable engravings, many of which are not to be met with elsewhere, it is a work which we think every dental student, and especially practitioner, should possess. The plates alone are worth the price of the book. We have no doubt, that should the publishers strike these off with their illustrations upon (say five) large sheets, to be framed and hung up in the dental offices, that they would meet with a ready sale. We should certainly be glad to procure them in this form. We shall take occasion to notice this work more at length in the next number of the Journal. W.

